# On-Orbit Relative Radiometric Calibration of the Night-Time Sensor of the LuoJia1-01 Satellite

**DOI:** 10.3390/s18124225

**Published:** 2018-12-02

**Authors:** Guo Zhang, Litao Li, Yonghua Jiang, Xin Shen, Deren Li

**Affiliations:** 1 State Key Laboratory of Information Engineering in Surveying, Mapping and Remote Sensing, Wuhan University, Wuhan 430079, China; guozhang@whu.edu.cn (G.Z.); xinshen@whu.edu.cn (X.S.); drli@whu.edu.cn (D.L.); 2 School of Remote Sensing and Information Engineering, Wuhan University, Wuhan 430079, China; jiangyh@whu.edu.cn

**Keywords:** LuoJia1-01, radiometric calibration, detector non-uniformity, nighttime sensor

## Abstract

The LuoJia1-01 satellite can acquire high-resolution, high-sensitivity nighttime light data for night remote sensing applications. LuoJia1-01 is equipped with a 4-megapixel CMOS sensor composed of 2048 × 2048 unique detectors that record weak nighttime light on Earth. Owing to minute variations in manufacturing and temporal degradation, each detector’s behavior varies when exposed to uniform radiance, resulting in noticeable detector-level errors in the acquired imagery. Radiometric calibration helps to eliminate these detector-level errors. For the nighttime sensor of LuoJia1-01, it is difficult to directly use the nighttime light data to calibrate the detector-level errors, because at night there is no large-area uniform light source. This paper reports an on-orbit radiometric calibration method that uses daytime data to estimate the relative calibration coefficients for each detector in the LuoJia1-01 nighttime sensor, and uses the calibrated data to correct nighttime data. The image sensor has a high dynamic range (HDR) mode, which is optimized for day/night imaging applications. An HDR image can be constructed using low- and high-gain HDR images captured in HDR mode. Hence, a day-to-night radiometric reference transfer model, which uses daytime uniform calibration to calibrate the detector non-uniformity of the nighttime sensor, is herein built for LuoJia1-01. Owing to the lack of calibration equipment on-board LuoJia1-01, the dark current of the nighttime sensor is calibrated by collecting no-light desert images at new moon. The results show that in HDR mode (1) the root mean square of mean for each detector in low-gain (high-gain) images is better than 0.04 (0.07) in digital number (DN) after dark current correction; (2) the DN relationship between low- and high-gain images conforms to the quadratic polynomial mode; (3) streaking metrics are better than 0.2% after relative calibration; and (4) the nighttime sensor has the same relative correction parameters at different exposure times for the same gain parameters.

## 1. Introduction

Nocturnal lighting is a primary method that enables to study human activity from space and is used extensively worldwide in residential, commercial, industrial, and public facilities and roadways [1]. Conventional daytime remote sensing is mainly focused on the observation of natural systems on Earth’s surface while night-time remote sensing is human-centered observation that directly reflects human activities. Having a capability for direct global observation of human activities that vary in intensity could substantially improve understanding of the magnitude of humanity’s presence and help in modelling human impacts on the environment. Satellite observation of the location and intensity of nocturnal lighting provides a unique view of humanity’s presence and can be used as a spatial indicator for other variables that are more difficult to observe on a global scale [1]. The remote sensing of night-time lighting has been studied and shown to be economical and straightforward for applications [2] such as extracting, assessing, and monitoring urbanization dynamics [3,4,5], power consumption [6], gas flaring volume [7], CO_2_ emissions [8], material stocks [9], analyzing urban activities based on street lights [10,11,12,13,14], recognition of fishing boats based on light used for fishing [15], investigating artificial light pollution [16,17,18,19], armed conflicts [20] and mapping urban extents [21].

The first professional nighttime remote sensing satellite, LuoJia1-01 (referred to as LJ1-01), was successfully launched on 2 June 2018. The nighttime light data products have great application potential and have been successfully applied in economics [19]. LJ1-01 is equipped with a 4-megapixel scientific CMOS Image Sensor. The sensor parameters as shown in Table 1.

The nighttime sensor of the LJ1-01 uses an electronic rolling shutter [22]. Each instantaneous exposure images one row of a single frame. The 2048 rows of detectors are exposed sequentially, and a full frame of data is recorded after all row detectors have been exposed. The time interval between the capture of two frames is the frame period; this can be adjusted for different missions. The sensor has two operation modes: the STD mode (STD), which operates at a frame rate of 48 fps, and the high dynamic range (HDR) mode, which is optimized for high dynamic range applications at a frame rate of 24 fps. In HDR mode, the sensor can capture a low- and high-gain image at each exposure. An HDR image can be constructed by the HDR image construction method. The sensor has a variety of imaging parameters, such as multiple gain level and exposure time settings, which can be adjusted for daytime and nighttime imaging. In HDR mode, the sensor uses a combination of low-level gain and short exposure time for daytime imaging. The low-gain image is effective and the high-gain image is saturated in daytime imaging scenarios. Low-level gain and long exposure time are used for nighttime imaging. In nighttime imaging scenarios, both the low and high-gain images are effective.

**Figure 1:**
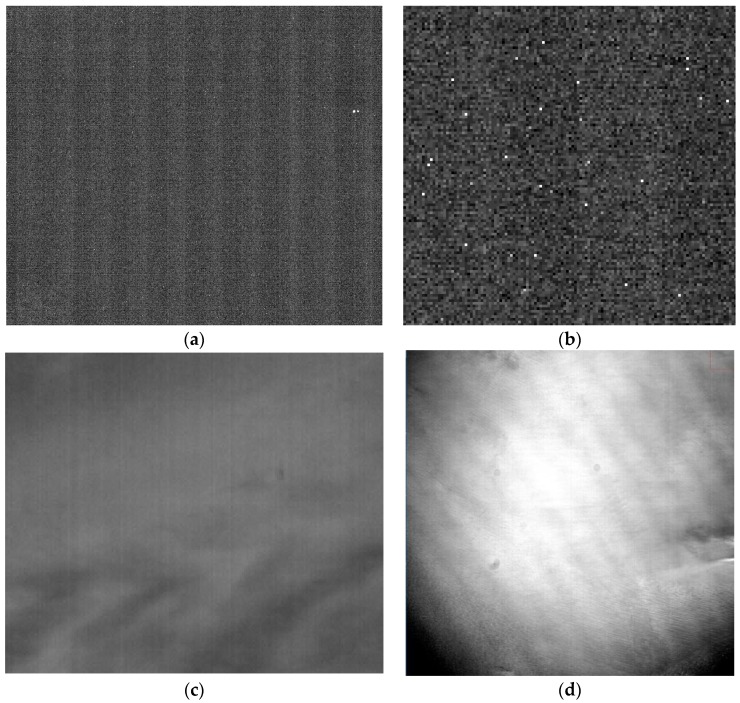
Some noticeable artifacts in raw images: (**a**) dark current striping, (**b**) hot pixels (zoomed in 4×), (**c**) non-uniformity striping, and (**d**) vignetting artifact.

The raw imagery collected by LJ1-01 exhibits some noticeable detector-level errors, such as striping from detector dark current (Figure 1a), hot pixels (Figure 1b), striping from detector non-uniformity (Figure 1c), and vignetting artifacts (Figure 1d).

Relative radiometric calibration can calibrate these detector-level errors, and is a prerequisite for the application of nighttime light data. Relative radiometric calibration can be divided into two stages: laboratory calibration and on-orbit calibration. Laboratory calibration [23] is used to calibrate all the performance parameters of the sensor prior to satellite launch, and is used to provide initial radiometric calibration results. Owing to mechanical stresses experienced during satellite launch as well as the influence of the environment in space, the response value of the sensor detectors will change with time. Thus, on-orbit calibration is required to ensure the imaging quality throughout the life cycle of the satellite sensor.

Conventionally, the radiometric response of the sensor detectors follows a linear model [24], as shown in Equation (1):

(1) DN(k,n,b,m,t)=A(k)∗G(m,t,k)∗γ(k,m,b)∗g(k,n,b)∗L(k,n,b)+C(k,n,b,m)

where *k* is the band index, *n* is the sensor detector index, *b* is the charge-coupled device (CCD) index, *m* is the gain parameter, *t* is the exposure time, and *L* is the input radiance. The parameters of the response model are divided into two categories: (1) relative calibration parameters (normalized parameters), which include dark current C(k,n,b,m), inter-detector non-uniformity g(k,n,b), and read-out register γ(k,m,b) coefficients; and (2) absolute calibration parameters, which include programmable amplification and exposure time G(m,t,k) and absolute calibration coefficient A(k). This study focuses on the on-orbit calibration of the relative calibration coefficient of LJ1-01, the read-out register *γ* = 1, since the nighttime sensor of LJ1-01 has one CMOS sensor.

A high precision calibration reference that can be used for all sensor detectors is needed to calibrate the relative calibration coefficients. Conventional optical remote sensing satellites that are used only for daytime imaging employ on-orbit calibration reference [25,26,27,28,29] (such as on-board light or solar diffusers), uniform Earth scenes [30,31,32] (such as deserts, oceans, deep convective clouds, and snow), and statistical references [33,34]. For a sensor with nighttime light imaging capabilities, the Visible Infrared Imaging Radiometer Suite (VIIRS) of Suomi-NPP is a nadir-viewing imaging sensor that uses a rotating telescope and accompanying optics to scan the Earth in the across-track direction, which is perpendicular to the direction of flight [35]. For the VIIRS sensor on board Suomi-NPP, statistical reference is used to calibrate the relative calibration coefficient of the three gain stages image of the Day-Night Band (DNB), the on-board solar diffuser is used to calibrate the ratio of the three gain stages image [36], and ocean data acquired at new moon are used to calibrate the sensor dark current [35]. Each frame of the LJ1-01 sensor represents 264 km × 264 km on the Earth’s surface. It is difficult to obtain a uniform nighttime light source of such a large area, and the existing statistical method cannot be used for LJ1-01. To overcome this challenge, the nighttime sensor of LJ1-01 was designed to have daytime and nighttime imaging capabilities, and the relative calibration is accomplished by building a day-night radiometric reference transfer model. Therefore, this paper presents the on-orbit relative calibration scheme of LJ1-01, which transfers the daytime reference to the night to realize the ‘daytime calibration and nighttime correction’.

## 2. Method

**Figure 2:**
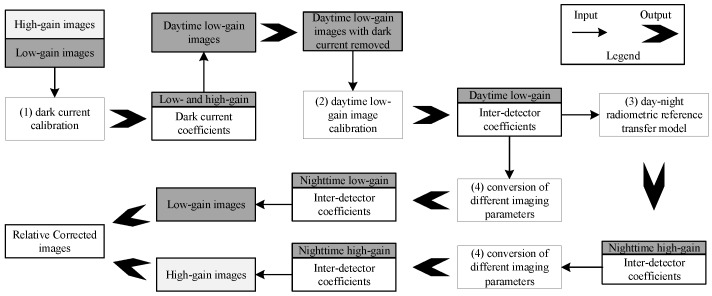
Flow chart of on-orbit relative calibration of the LJ1-01 nighttime sensor in HDR mode.

Using the daytime imaging capability of LJ1-01, we transfer the daytime calibration to the nighttime. The on-obit calibration process includes: (1) calibrating dark current, (2) calibrating daytime low-gain images, (3) building the day-night radiometric reference transfer model, and (4) converting different imaging parameters, as shown in Figure 2. Then, the low and high-gain images are corrected using the relative calibration coefficients, and the HDR image can be obtained by the HDR construction method.

### 2.1. Dark Current Calibration

Dark current calibration is used to calibrate the sensor response value when there is no input radiance. The nighttime sensor of LJ1-01 has a high sensitivity and lacks on-board calibration payload, such as the shutter of optical remote sensing satellites. On-orbit dark current calibration of LJ1-01 is performed by imaging the uniform no-light Sahara Desert or deep ocean at new moon. The dark current calibration model is shown in Equation (2):

(2) $$C_{i}=\frac{1}{M}\sum_{j}^{M}DN_{i,j}$$

where *i* is the sensor detector index, *j* is the valid frame index, *M* is the valid frame number, $C_{i}$ is the dark current value of the *i*th detector, and $DN_{i,j}$ is the valid DN after eliminating the gross error with 5 DN as the threshold. The dark current of all detectors is corrected to the reference value $\bar{C}$, using Equation (3), as follows:

(3) $$\bar{C}=\sum_{i}^{N}C_{i}$$

where *N* is the detector number (*N* = 2048 × 2048 for LJ1-01). Considering the absolute radiometric coefficients of the laboratory calibration of LJ1-01, the on-orbit dark current correction equation is:

(4) $$DN_{c,i}=DN_{i}-C_{i}+\bar{C}$$

where $DN_{i}$ is the DN of the *i*th detector, and $DN_{c,i}$ is the corrected DN of the *i*th detector. See Appendix A for the detailed processing in pseudocode for the dark current calibration of the LJ1-01 nighttime sensor.

### 2.2. Daytime Calibration of Low-Gain Images

The linear model (Equation (1)) is used to calibrate the inter-detector coefficients, based on a uniform daytime Earth scenes. The input radiance *L* can be replaced by the detectors’ mean $\bar{DN}$ in the relative calibration, and Equation (1) can be transformed to Equation (5):

(5) $$\bar{DN}=a_{i}*DN_{c,i}+b_{i}$$

where $\bar{DN}$ is the mean of all detectors, and $a_{i}$ and $b_{i}$ are the relative coefficients of the *i*th detector of the sensor. However, all detectors of the nighttime sensor of LJ1-01 cannot be covered by all uniform calibration scenes and cannot be calibrated simultaneously. Some detectors can be directly calibrated, and the calibration of other detectors is achieved using the inter-detector relationship. When all detectors of the sensor image a uniform scene, the DN variations in the data acquired by the sensors are caused by the non-uniformity in the responses of the detectors. When the relative coefficient $b_{i}$ is ignored, the response difference coefficient $g_{i}$ of each detector is given by:

(6) $$g_{i}=\frac{\bar{DN}}{DN_{c,i}}$$

Set the relative correction model of the *i*th detector and the reference detector *j* as:

(7) $$\bar{DN}=a_{i}*DN_{i}+b_{i}$$

(8) $$\bar{DN}=a_{j}*DN_{j}+b_{j}$$

By Equation (6), we have:

(9) $$\frac{DN_{i}}{DN_{j}}=\frac{g_{j}}{g_{i}}$$

Combining Equations (7)–(9), the relative calibration coefficients $a_{i}$ and $b_{i}$ are:

(10) $$a_{i}=\frac{g_{i}}{g_{j}}*a_{j}$$

(11) $$b_{i}=b_{j}$$

Thus, the relative correction equation of LJ1-01 is:

(12) $$DN_{i}^{relCorr}=(DN_{i}-C_{i})*a_{i}+b_{i}+\bar{C}$$

where $DN_{i}^{relCorr}$ is the corrected DN of the *i*th detector.

### 2.3. Day-Night Radiometric Reference Transfer

For daytime imaging in HDR mode, the low-gain images are effective, while the high-gain images are saturated. However, for nighttime imaging, both low and high-gain images are effective. Therefore, after the calibration of daytime low-gain images is completed, the daytime calibration reference needs to be transferred to the nighttime images in order to correct the nighttime low and high-gain images. From Equation (5), the relative correction for LJ1-01 is:

(13) $$\bar{DN_{high}}=a_{high}*DN_{high}+b_{high}$$

(14) $$\bar{DN_{low}}=a_{low}*DN_{low}+b_{low}$$

where $a_{high}$, $b_{high}$, $a_{low}$, and $b_{low}$ are the relative calibration coefficients of the high and low-gain images, $DN_{high}$ and $DN_{low}$ are the original DNs of the high and low-gain images, and $\bar{DN_{high}}$ and $\bar{DN_{low}}$ are the correction references of the high and low-gain images, respectively.

The high/low-gain conversion model of the LJ1-01 nighttime sensor was derived from the high/low-gain signal model of the sensor in satellite design [37,38]. The high-gain images are obtained by on-board gain control based on the low-gain image signals. The sensor gain control is theoretically composed of three parts: the pixel output amplifier gain, which is generated by the MOSFET structure as a trans-impedance amplifier; the signal processing unit gain $A_{1}$; and the analog/digital converter (ADC) gain $A_{2}$. The output signal, S(DN), that results for a given exposure period is given by:

(15) $$S\left(DN\right)=P\cdot QE_{I}\cdot \eta_{i}\cdot S_{v}\cdot A_{CCD}\cdot A_{1}\cdot A_{2}$$

where S(DN) represents the average signal for a group of pixels (DN), P is the average number of incident photons per pixel, $QE_{I}$ is the interacting QE, $\eta_{i}$ is the quantum yield, $S_{v}$ is the sensitivity of the sensor node, $A_{CCD}$ is the output amplifier gain, $A_{1}$ is the gain of the signal processor, and $A_{2}$ is the gain of the ADC. Among them, the pixel output amplifier gain changes significantly with the light intensity [39,40], which causes the high/low-gain conversion model of the sensor to be non-linear. Therefore, the DN model relationship between low- and high-gain images is not a linear model in the quantization range and the real model and model parameters can be obtained by fitting the preflight calibration data. Based on theory, an unknown-order polynomial model is chosen as the model relationship between the low- and high-gain DNs of LJ1-01 in HDR mode, as shown in Equation (16):

(16) $$DN_{high}=B_{0}+B_{1}*DN_{low}+B_{2}*DN_{low}{}^{2}+\ldots +B_{n}*DN_{low}{}^{n}$$

(17) $$\bar{DN_{high}}=B_{0}+B_{1}*\bar{DN_{low}}+B_{2}*{\bar{DN_{low}}}^{2}+\ldots +B_{n}*{\bar{DN_{low}}}^{n}$$

where *n* is a polynomial order, and $\;B_{0}$, $B_{1}$, $B_{2}$, $B_{n}$ are polynomial coefficients.

Because the model between the low and high-gain DNs is not a linear model, $DN_{high}$ and $\bar{DN_{high}}$ do not conform to the linear model, shown in Equation (13), and the model between $DN_{high}$ and $\bar{DN_{high}}$ needs to be derived from Equations (14)–(17). When *n* = 2, the model relationship between $DN_{high}$ and $\bar{DN_{high}}$ is:

(18) $$\begin{array}{cc} \bar{DN_{high}}= & \frac{{\left(B_{1}\pm \sqrt{-4B_{2}B_{0}+B_{1}^{2}+4B_{2}DN_{high}}\right)}^{2}a_{low}^{2}}{4B_{2}}\\ & \;\;-\frac{\left(2B_{2}b_{low}a_{low}+B_{1}a_{low}\right)\left(B_{1}\pm \sqrt{-4B_{2}B_{0}+B_{1}^{2}+4B_{2}DN_{high}}\right)}{2B_{2}}\\ & \;\;+B_{2}b_{low}^{2}+B_{1}b_{low}+B_{0} \end{array}$$

See Appendix A for the detailed processing in pseudocode for the relative calibration of the LJ1-01 nighttime sensor.

### 2.4. Conversion of Calibration Coefficients under Different Imaging Parameters

The nighttime sensor of LJ1-01 is a high-sensitivity sensor that uses a combination of different daytime and nighttime imaging parameters in HDR mode to ensure that the daytime imagery is not saturated, and that the brightness of the nighttime imagery light is within a reasonable DN range. The detector correction model relationship under different imaging parameters needs to be considered. As shown Table 2, the daytime and nighttime imaging parameters of LJ1-01 in HDR mode only have different exposure times. Therefore, it is necessary to ascertain the correction model relationship for different exposure times.

According to the principle of remote sensing satellite sensor design [37], the DN recorded by the sensor is proportional to the gain multiplier *g* and the exposure time *t*. Considering the detector dark current $c_{0}$, we have:

(19) $$DN\left(g,t\right)\propto g*\left(t*L+c_{0}\right)$$

where *L* is the input radiance, and  DN(g,t) is the DN recorded by the sensor. From Equation (19), it can be seen that the radiance difference caused by different exposure times can be considered in the absolute calibration coefficients. Therefore, the sensor has the same relative correction parameters at different exposure times.

## 3. Experiment and Results

### 3.1. Data Introduction

The ground resolution of LJ1-01 is 129 m, and the large uniform calibration Earth scene (264 km × 264 km) that satisfies the sensor non-uniformity calibration requirements and covers all detectors of the LJ1-01 sensor is relatively rare. Markham et al. [41] pointed out that when the non-uniformity calibration accuracy is better than 0.5%, the non-uniformity caused by the uniform Earth scene must be better than 0.05%. The principle of selecting a large uniform Earth scene for on-orbit calibration is based on the experience of Basnet [42] and Gerace [43], and uses the large uniform scenes defined by Landsat 5 images on a global scale. The selected uniform scenes are listed in Table 3.

From July to August 2018, many calibration missions were performed, but the only one dark current calibration data and three non-uniformity calibration data were available. The available mission information is listed in Table 4. Data from about fifty-eight frames were used to verify the dark current calibration results, and the non-uniformity calibration verification data consisted of full clouds illuminated by moonlight and partial nighttime light data.

### 3.2. Results of Dark Current Calibration

**Figure 3:**
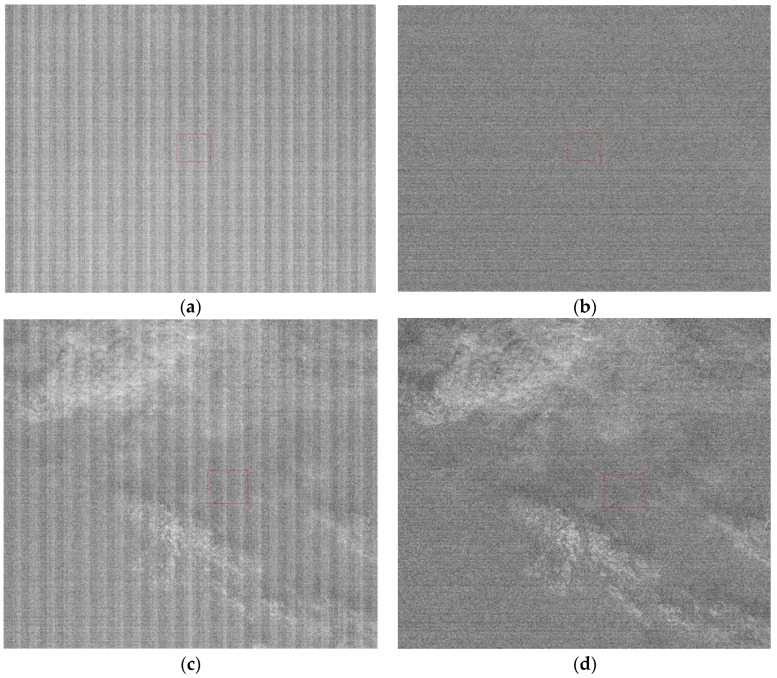
High- and low-gain dark current correction results for LJ1-01. (**a**) Low-gain original data without light, (**b**) low-gain dark current corrected data, (**c**) high-gain original data without light, and (**d**) high-gain dark current corrected data.

Fifty-six dark current frames were used to calibrate the low and high-gain dark current response of the nighttime sensor of LJ1-01 in HDR mode. The low (high) gain dark current mean of all detectors was 187.31 (177.57). This shows that the nighttime sensor of LJ1-01 has high-sensitivity. The dark current calibration coefficients are applied to the nighttime images, as shown in Figure 3. The difference in the high and low-gain dark current response of all detectors is effectively corrected, and the hot pixels are also removed.

For quantitative analysis, the root mean square of the mean for each detector was used to estimate the residual error after dark current correction, as shown in Equation (20):

(20) $$\delta =\sqrt{\frac{1}{N}{\left({\bar{DN}}_{i}-\bar{DN}\right)}^{2}}$$

where δ is the residual error after dark current correction, *N* is the detector number, $DN_{i}$ is the mean value of the *i*th detector in the row direction of the sensor, and $\bar{DN}$ is the mean value of all detectors.

Fifty-eight frames with dark current removed were used for statistical calculation based on Equation (20), as shown in Table 5. The root mean square of the mean for each detector in the low (high) gain image is better than 0.04 (0.07) DN.

### 3.3. Daytime Low-Gain Calibration

**Figure 4:**
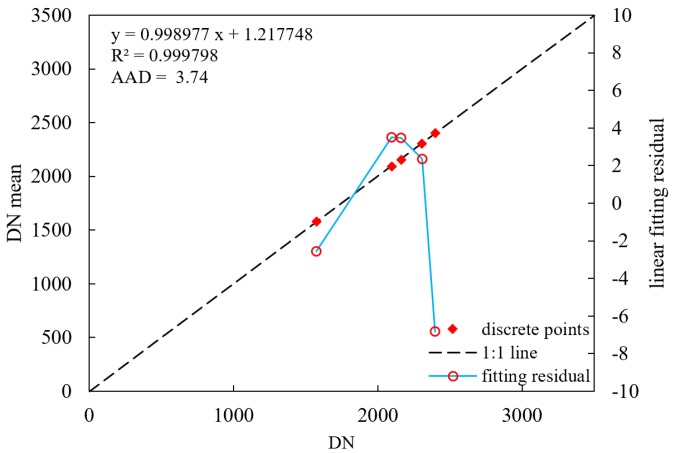
The linear model and fitting residual of the reference detector of nighttime sensor.

**Figure 5:**
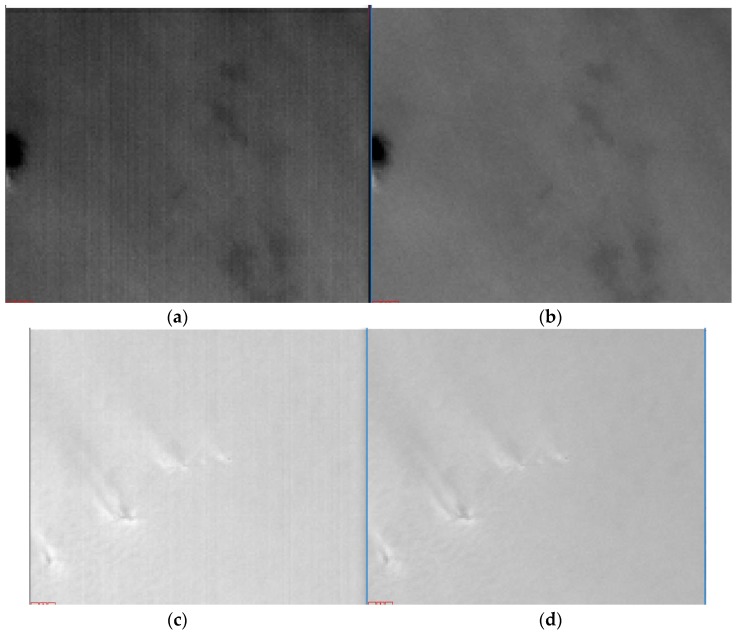
Non-uniformity correction results of daytime low-gain images (zoomed in 4×). (**a**) and (**c**) are the original images; (**b**) and (**d**) are the relative corrected images.

**Figure 6:**
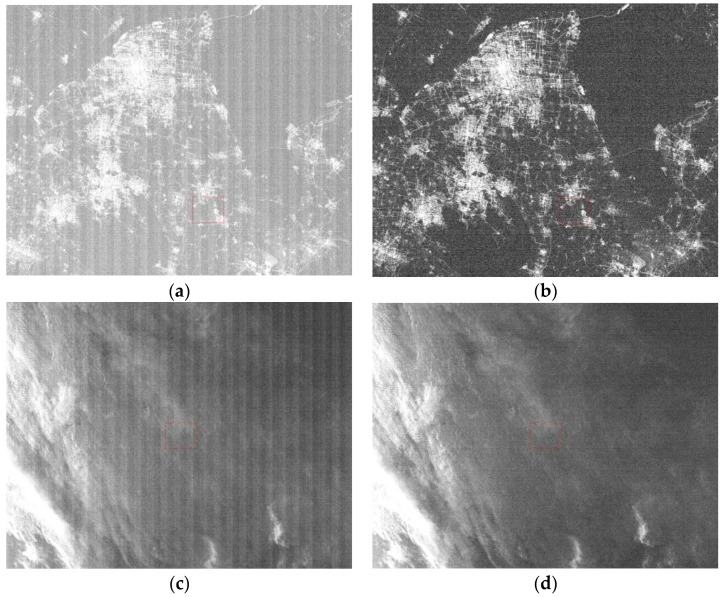
Non-uniformity correction results of nighttime low-gain images. (**a**) and (**c**) are the original images; (**b**) and (**d**) are the relative corrected images.

The Arabia 2 uniform data were used to calculate the detector response difference coefficients based on Equation (6). The same zone (9 × 9) in the five uniform data was selected to calculate the relative calibration coefficients, and the relative coefficients of the rest of the LJ1-01 detectors were obtained based on Equations (10) and (11). As shown in Figure 4, the R-square of linear model of the reference detector achieves 99.98%, the maximum residual is better than 7 DN, and the average absolute difference (AAD) is 3.74. Since the light image cannot cover all sensor detectors, it is difficult to distinguish the correction results. Therefore, the day/night terminator images were selected to verify the calibration results. The strips caused by the differences in inter-detector response were eliminated, as shown in Figure 5 and Figure 6.

Because the foremost goal of relative calibration is to eradicate detector-to-detector non-uniformity (streaking), a streaking metric is herein used as the primary means of comparison. In a uniform data product, a detector is compared to its immediate neighbors using Equation (21):

(21) $$Streaking_{i}=\frac{\left|\bar{DN_{i}}-\left(\bar{DN_{i-1}}+\bar{DN_{i+1}}\right)/2\right|}{\left(\bar{DN_{i-1}}+\bar{DN_{i+1}}\right)/2}\times 100$$

where $\bar{DN_{i}}$ is the mean value of the *i*th detector in the row direction of the sensor. Streaks become visibly apparent in homogeneous unaltered imagery when the streaking metric is approximately 0.25% [28].

**Figure 7:**
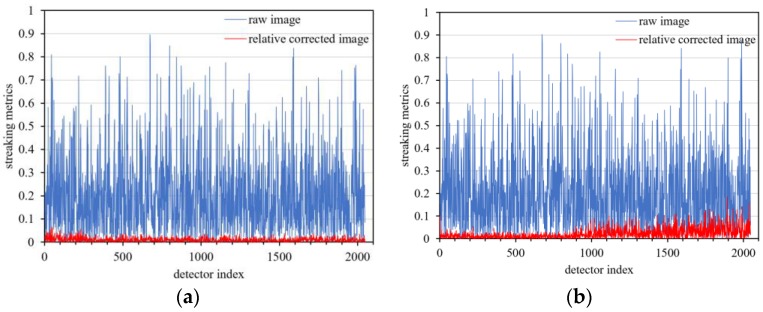
Streaking metrics of daytime low-gain images after relative correction. (**a**) Mauritania 2 uniform data, (**b**) Niger 2 uniform data.

The relative corrected daytime data and the day/night terminator data were calculated based on Equation (21). As shown in Figure 7, the maximum streaking metrics of relative corrected daytime images are better than 0.17% and 0.18%.

**Figure 8:**
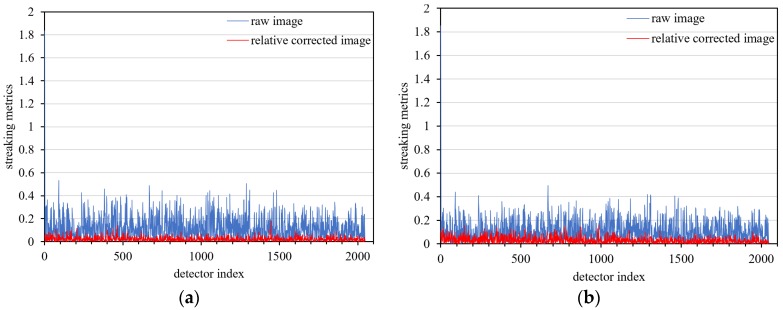
Streaking metrics of nighttime low-gain images after relative correction. (**a**) uniform clouds data illuminated by moonlight, (**b**) day/night terminator data.

For nighttime images, the maximum streaking metrics of relative corrected images are better than 0.18% and 0.16% (Figure 8).

### 3.4. Day-Night Radiometric Reference Transfer

#### 3.4.1. Parameter Solving for the High-Gain and Low-Gain Response Model

The highest order and model parameters of the high-gain and low-gain response polynomial model are determined using the preflight calibration data and on-orbit data.

##### Preflight Calibration Data

**Figure 9:**
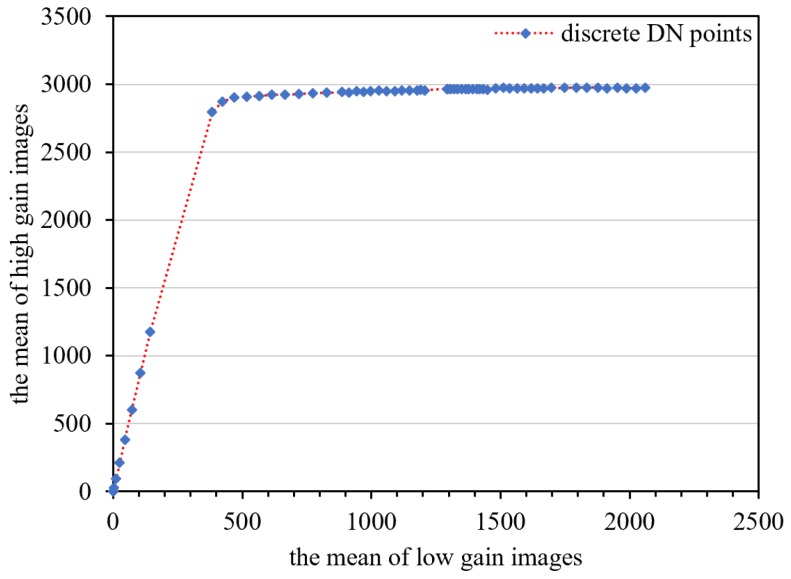
Response relationship between the low- and high-gain means of laboratory calibration data.

**Figure 10:**
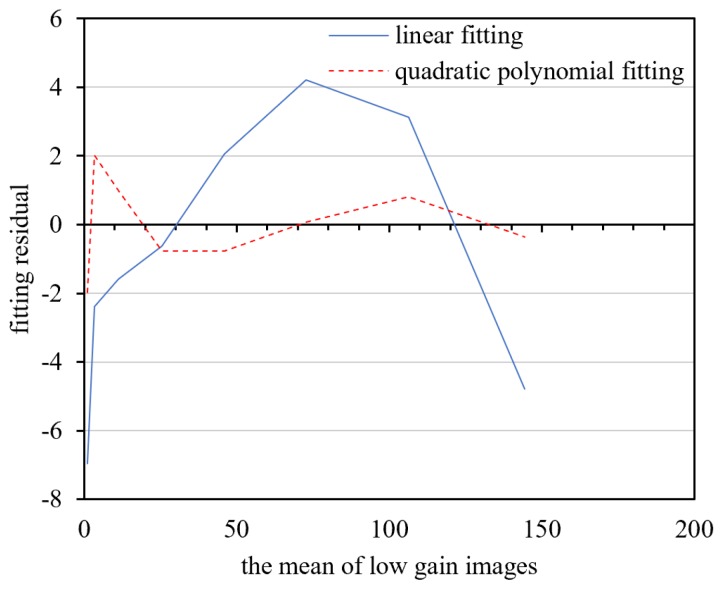
Fitting residual errors of middle-radiance points of high- and low-gain images.

Sixty-eight sets of original calibration data under different input radiances were selected to establish the response model relationship between the mean value of the low-gain images and the that of the high-gain images after dark current correction. As shown in Figure 9, a linear model is discernable in the eight middle-radiance points. As shown in Figure 10, the linear and polynomial models were used to fit the eight middle-radiance points. The fitting residuals indicate that the response relationship between the DNs of the high and low-gain images is closer to the second-order polynomial model. The model DN range is [0.9,382.9] for low-gain images, and [2.9,2793] for high-gain images. This means that the high/low-gain DN of nighttime imaging is mostly within the range of the second-order polynomial model. There are 52 DN points in the high-radiance range, which is also consistent with the second-order polynomial model.

##### On-Orbit Data

**Figure 11:**
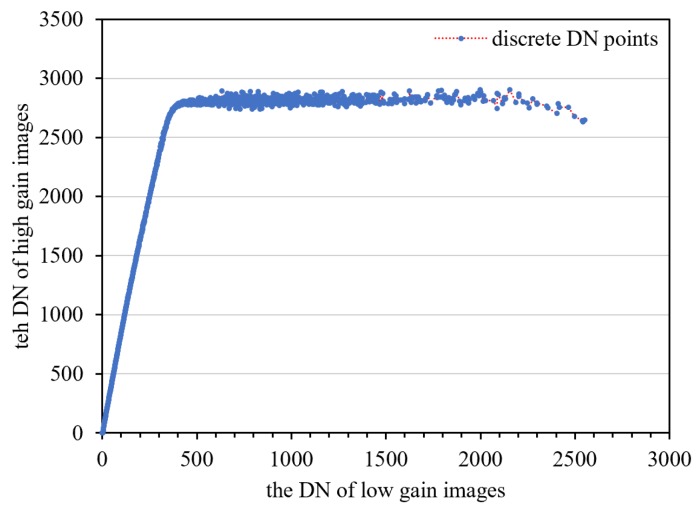
The high/low-gain DN response relationship presented by on-orbit data.

**Figure 12:**
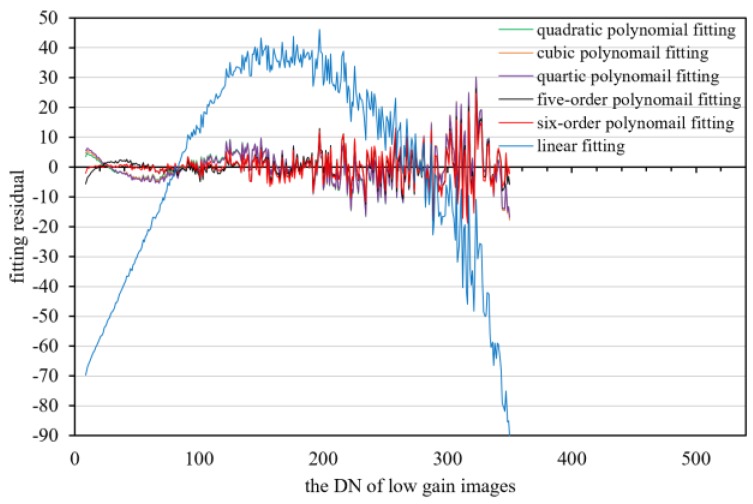
The fitting residual errors of middle-radiance points of high and low-gain images under different polynomial order.

Data from 1396 frames with the same imaging parameters in HDR mode were counted, and the high/low-gain DN relationship is shown in Figure 11. The second- to sixth-order polynomials are used to fit the middle-radiance points. The fitting residuals from the second- to sixth-order polynomial are basically equal, and the fitting residuals do not decrease as the fitting order increases Figure 12. This indicates that the high/low-gain response model in HDR mode is sufficiently expressed with a second-order polynomial model.

By analyzing the results obtained from preflight data and on-orbit data, we confirm that (1) the cut-off between the middle-radiance points and high-radiance points is approximately [380,2800]; (2) the optimal fitting model of the high/low-gain response model in HDR mode is a second-order polynomial model.

##### Model Parameter-Solving

The polynomial model between the high and low-gain DNs of LJ1-01 was solved using the least square fit method for the polynomial coefficients. A set of model parameters fitted using preflight calibration data is shown in Table 6.

#### 3.4.2. Nighttime Correction

**Figure 13:**
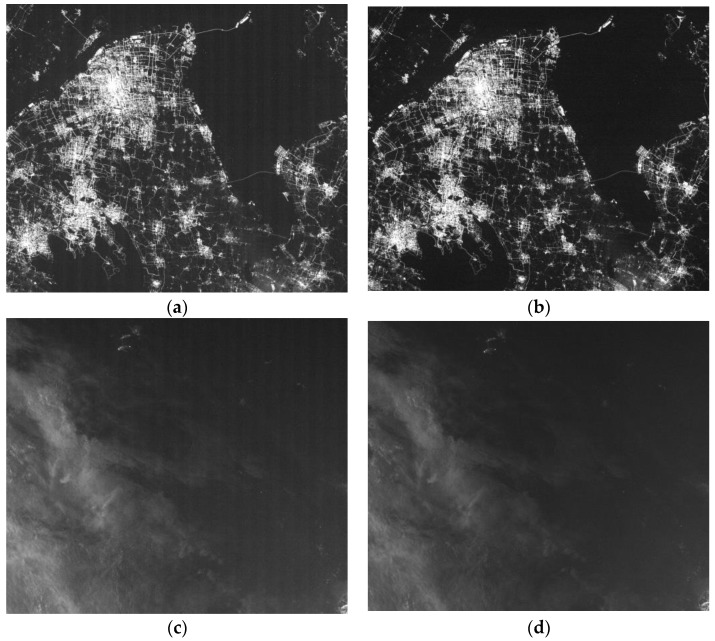

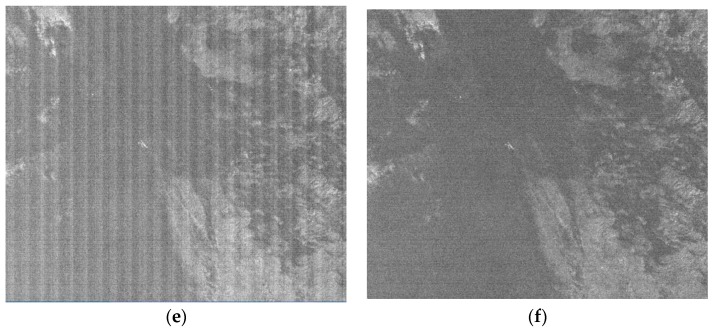
Results of relative correction of nighttime high-gain images. Original images of (**a**) city lights, (**c**) day/night terminator, and (**e**) clouds illuminated by moonlight. Relative corrected images of (**b**) city lights, (**d**) day/night terminator, and (**f**) clouds illuminated by moonlight.

The relative calibration coefficients of the low-gain DNs calibrated by daytime uniform calibration were converted to the relative calibration coefficients of the nighttime high-gain DNs based on the day-night radiometric reference transfer model, as shown in Equation (21). The converted relative calibration coefficients were applied to the nighttime images at high gain. The converted relative calibration coefficients achieve good correction effects on the high-gain images of different brightness scenes, such as city lights, day/night terminator, and uniform clouds illuminated by moonlight (Figure 13).

The nighttime sensor of LJ1-01 is an array image sensor and the collected images will still exhibit vignetting (i.e., the ‘middle bright edge dark’ effect) caused by the lens. The compensation effect of the vignetting artifact was checked by calculating the mean value of the along-track and across-track direction of the uniform cloud images. 

**Figure 14:**
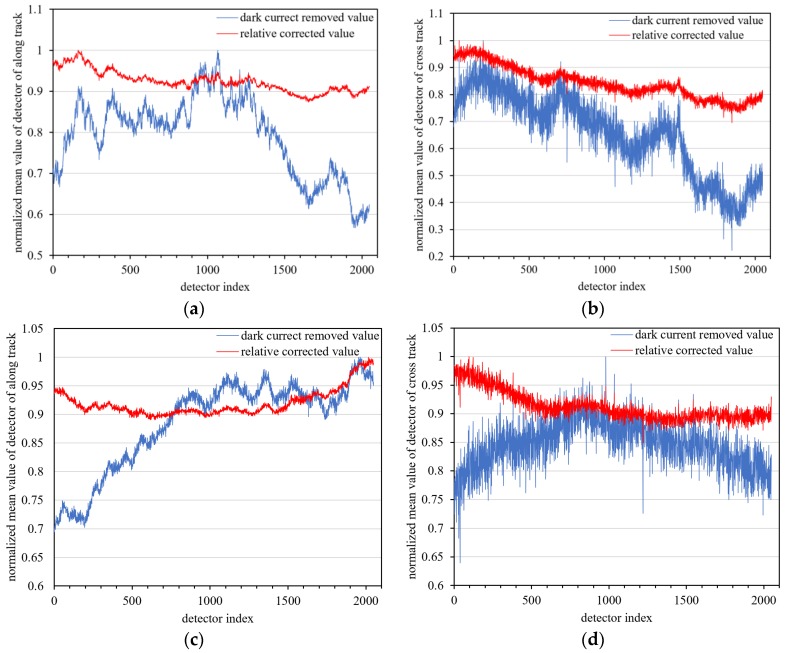
Vignetting compensation effect for high gain in the along-track (**a**,**c**) and across-track (**b**,**d**) direction between relative corrected images and images with only dark current removed.

As shown in Figure 14, compared with the images after only dark current correction, there is effective compensation for the vignetting artifact in the along-track and across-track directions in the relative corrected images, indicating that the high-gain relative coefficients are validated by the day-night radiometric reference transfer model.

**Figure 15:**
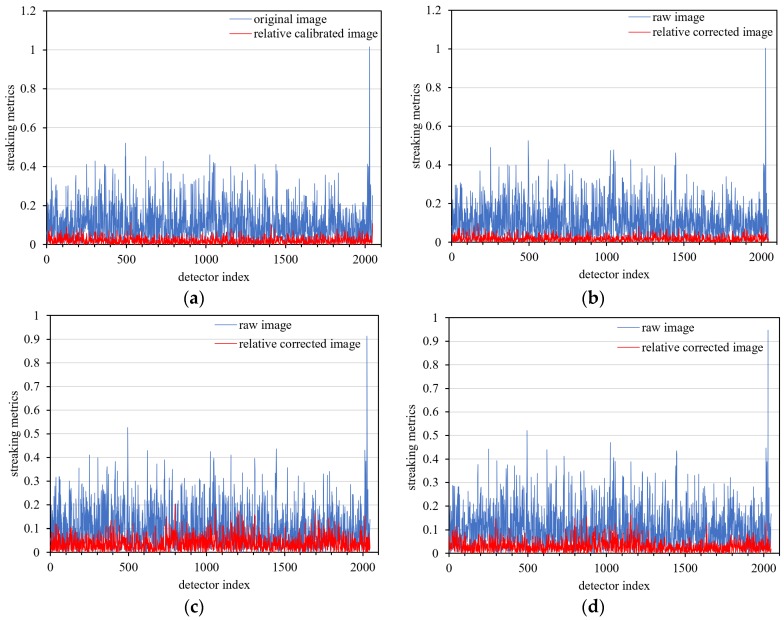
Streaking metrics of four uniform nighttime high-gain cloud scenes with relative correction. (**a**) zone 1 uniform data, (**b**) zone 2 uniform data, (**c**) zone 3 uniform data, (**d**) zone 4 uniform data.

For quantitative analysis, the streaking metrics (as shown in Equation (21)) of four regions for the uniform cloud data product were calculated to estimate the accuracy of relative correction. As shown in Figure 15 and Table 7, the maximum streaking metrics of relative corrected nighttime high-gain images is better than 0.2%, indicating that the ‘daytime calibration and nighttime correction’ scheme is effective and can yield good correction results.

### 3.5. Validating Different Correction Coefficients under Different Imaging Parameters

**Figure 16:**
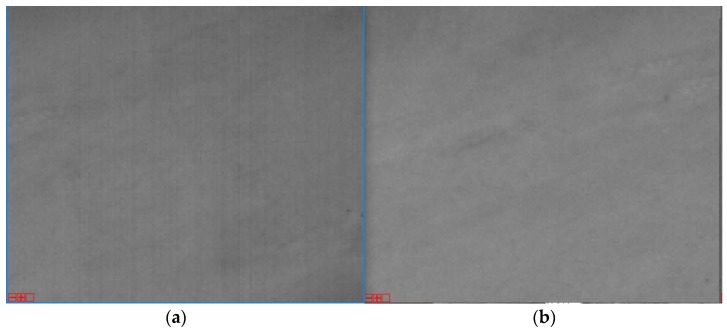
Relative correction results of different exposure times (zoomed in 4×). (**a**) Daytime low-gain image at an exposure time of 0.049 ms, and (**b**) corrected image at an exposure time of 18.8 ms.

The preflight calibration data were used to verify the validity of the correction coefficients at different exposure times for the same gain parameters. The correction obtained for the exposure time of 0.049 ms were used to correct the data for the exposure time of 18.8 ms. The results are shown in Figure 16. This indicates that the relative correction coefficients are not affected by changes in exposure time.

## 4. Discussion

**Figure 17:**
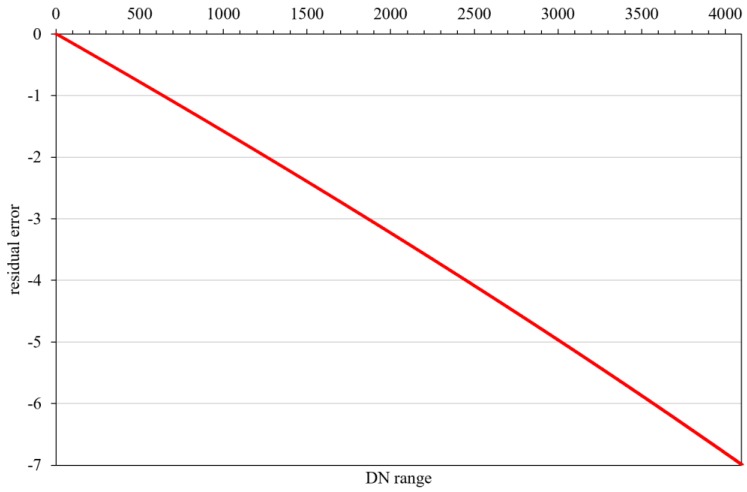
Fitting residual of linear and nonlinear high-gain model of LJ1-01.

The day-night radiometric reference transfer model (that is high/low-gain conversion model) of LJ1-01 is nonlinear. Based on this, the converted high-gain relative correction model is very complex, as shown in Equation (18). However, the conventional detection correction model is a mostly linear model. When we treat the high-gain relative correction model as a linear model (as shown in Equation (13)), the fitting residual of the two correction models within the sensor quantization range is less than 7 DN, as shown in Figure 17. This shows that even the high/low-gain conversion model is a nonlinear model. The nonlinear part of the high correction model is discarded, and the errors introduced by the linear correction model for the high-gain images do not exceed 7 DN. The response model of the sensor in the quantization range is still a linear response model.

The conventional process of converting photons collected by the sensor detectors into a digital signal quantified to DN during an exposure period is described as follows. Firstly, the incident radiance received by the sensor is transformed into continuous optical signal through optical system, then the optical signal is further converted into electrical signal. The signal amplified and processed by signal processing circuit is sampled by ADC and quantified to DN which expresses the image pixel information. The night-time sensor of LJ1-01 adopts dual readout sampling transmission mode in HDR mode to realize the low-high imaging function. However, the pixel output amplifier gain of the night-time sensor of LJ1-01 changes significantly with the light intensity, which causes the high/low gain conversion model of the sensor to be non-linear. The model parameters are empirical parameters obtained based on strict laboratory tests. As shown in Figure 10, the maximum fitting residual of the high-low gain model is less than 2 DN by using the preflight calibration data. Combining Equations (13) and (18), the corrected DN $DN_{high}^{relCorr}$ of the high gain images is given by:

(22) $$DN_{high}^{relCorr}=a_{high}*(DN_{high}\pm 2)+b_{high}=\bar{DN_{high}}\pm 2*a_{high}$$

The maximum correction error of high gain images comes from the conversion model does not exceed $2a_{high}$ DN when the high gain images are linear corrected, that is, the high gain images correction error does not exceed $\left(\frac{2a_{high}}{\bar{DN_{high}}}\cdot 100\right)$% at the maximum fitting residual.

## 5. Conclusions

In this work, the difficulty in the on-orbit relative radiometric calibration of the nighttime sensor of LJ1-01 without a light calibration reference is overcome by constructing a day-night radiometric reference transfer model. The schemes of ‘daytime calibration and nighttime correction’ is realized based on the capability of daytime imaging and the HDR imaging mode. The results show that the night-time sensor of LJ1-01 has a high sensitivity and good imaging quality in the HDR mode:

1) The root mean square of the mean for each detector in low (high) gain images is better than 0.04 (0.07) DN after dark current correction.
2) The DN relationship between the low and high-gain image conforms to the quadratic polynomial mode.
3) The streaking metrics are better than 0.2%, after relative calibration.
4) The nighttime sensor has the same relative correction parameters at different exposure times for the same gain parameters.

This study provides an effective method of on-orbit relative radiometric calibration of nighttime light remote sensing sensors, especially for planar array sensors. The conclusions obtained can help to further apply and research LJ1-01 products and contribute to researchers’ understanding of LJ1-01 nighttime light imagery. However, it was found that raw images from LJ1-01 have random stripes in the row direction under dark conditions. There are signs that this is aggravated with time, which further exacerbates the difficulty of on-orbit calibration. Addressing this problem remains for future research of on-orbit calibration of the LJ1-01 nighttime sensor.

## Figures and Tables

**Table 1:** Sensor parameters of LJ1-01.

Sensor Parameter	Value
Number of active detectors	2048 × 2048
Detector size	11 µm × 11 µm
Imaging mode	Standard (STD) mode High dynamic range (HDR) mode
Spectral range	460–980 nm
Resolution	129 m
Shutter type	Electronic rolling shutter
Quantization bits	12-bit, processing to 15bit @HDR mode
Frame rate	24 fps @HDR mode 48 fps @STD mode

**Table 2:** Daytime and nighttime imaging parameters of LJ1-01 in HDR mode.

	Daytime	Nighttime
Exposure times (ms)	0.049	17.089
Gain multiplier	0.6	0.6

**Table 3:** Uniform scenes for LJ1-01 on-orbit calibration.

Pseudo-Invariant Calibration Sites	Latitude (Degree)	Longitude (Degree)
Arabia 2, Middle East	20.24	51.03
Niger 2, Sahara	21.36	10.59
Mauritania 2, Sahara	20.23	−8.77
Egypt 2, Sahara	22.94	28.79

**Table 4:** On-orbit calibration mission information.

Calibration Mission	Imaging Time/Gain and Exposure Time	Imaging Region	LJ1-01 Images	Google Maps and Locations
Dark current calibration	20 July 2018/0.6 + 17.089 ms	Mauritania 2, Sahara	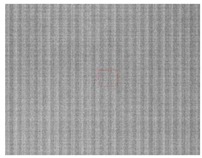	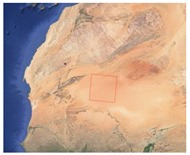
Non-uniformity calibration	20 July 2018/0.6 + 17.089 ms	Egypt 2, Sahara	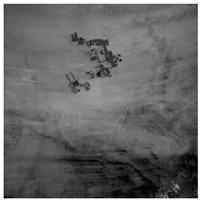	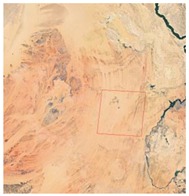
	22 July 2018/0.6 + 17.089 ms	Mauritania 2, Sahara	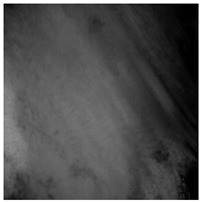	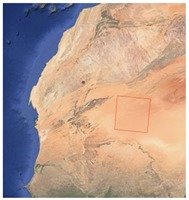
	23 July 2018/0.6 + 17.089 ms	Niger 2, Sahara	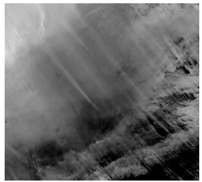	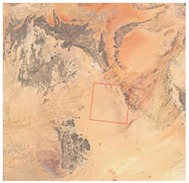
	18 August 2018/0.6 + 17.089 ms	Arabia 2, Middle East	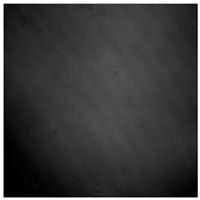	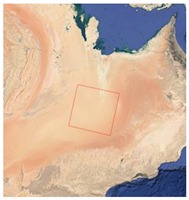

**Table 5:** Accuracy of dark current calibration of LJ1-01.

	Mean (DN)	Maximum (DN)	Minimum (DN)	Std. (DN*)*
Low gain	186.8194	186.9709	186.6585	0.041482
High gain	176.5858	176.8145	176.4118	0.066167

**Table 6:** A set of model parameters fitted using prefight calibration data.

	$B_{0}$	$B_{1}$	$B_{2}$	Goodness of Fit (R^2^)
Middle-radiance range	−3.046475	8.428720	−0.001721	0.999992
High-radiance range	2851.017690	0.132141	−0.000036	0.979314

**Table 7:** Relative correction accuracy of nighttime high-gain images of LJ1-01.

	Streaking Metrics (%)			
	Mean	Maximum	Minimum	Std.
Zone 1	0.021093	0.111714	0.0	0.016453
Zone 2	0.018854	0.097228	0.0	0.014664
Zone 3	0.030947	0.162012	0.0	0.024042
Zone 4	0.039596	0.202831	0.0	0.030460

## Appendix A

The detailed processing in pseudocode for the dark current calibration of the LJ1-01 nighttime sensor is as follows:

**Algorithm 1:** Description of the dark current calibration of the LJ1-01 nighttime sensor
**Input:**$DN_{i,j}{}^{org}$ from dark current calibration data includes the low- and high-gain images, i = 1, 2, 3, … N, j = 1, 2, 3, … M
**Output**: the dark current calibration results $C_{i}$ and $\bar{C}$
**for** each j = 1 to M do
Compute the average value of each frame of data:
$Mean_{j}=\sum_{i}^{N}DN_{i,j}{}^{org}$
**end for**
Eliminate the gross error and compute the valid $DN_{i,j}$:
$DN_{i,j}=DN_{i,j}{}^{org},\left|DN_{i,j}{}^{org}-Mean_{j}<5\right|$
**for** each i = 1 to N do
Compute the dark current value of each detector:
$C_{i}=\frac{1}{M}\sum_{j}^{M}DN_{i,j}$
**end for**
Compute the dark current correction reference:
$\bar{C}=\sum_{i}^{N}C_{i}$
Use the calibration results $C_{i}$ and $\bar{C}$ to correct the $DN_{i}$ of each single frame:
$DN_{c,i}=DN_{i}-C_{i}+\bar{C}$
**return**$C_{i}$, $\bar{C}$.

The detailed processing in pseudocode for the relative calibration of the LJ1-01 nighttime sensor is as follows:

**Algorithm 2:** Description of the relative calibration of the LJ1-01 nighttime sensor
**Input**: dark current calibration results $C_{i}$, i = 1, 2, 3, … N **Input:** dark current correction reference $\bar{C}$
**Input**: daytime low-gain calibration data $DN_{i,j}{}^{org}$, j = 1, 2, 3, … M **Input**: model relationship between low- and high-gain DNs of LJ1-01 in HDR mode, $P1\left[DN_{low},DN_{high}\right]$, $P2\left[\bar{DN_{low}},\bar{DN_{high}}\right]$
**Output**: relative calibration coefficients $a_{i,low}$ and $b_{i,low}$ for low-gain images and relative correction model for high-gain images $P3\left[\bar{DN_{high}},DN_{high}\right]$
**for** each j = 1 to M do for each i = 1 to N do
Correct the dark current:
$DN_{i,j}=DN_{i,j}{}^{org}-C_{i}+\bar{C}$
**end for**
**end for**
Compute the average values of all detectors for the uniform daytime calibration data (j = 1):
$\bar{DN}=\sum_{i}^{N}DN_{i,1}$
**for** each i = 1 to N do
Compute the response difference coefficient $g_{i}$:
$g_{i}=\frac{\bar{DN}}{DN_{i,1}}$
**end for**
Compute the average value of the interest zone (9 × 9) of each frame: ${\bar{DN}}_{j}$ Compute the reference detector relative calibration coefficients: $a_{ref}$ and $b_{ref}$
${\bar{DN}}_{j}=a_{ref}*DN_{ref,j}+b_{ref}$
**for** each i = 1 to N do
Compute the relative calibration coefficients of the other detectors for low-gain images:
$a_{i,low}=\frac{g_{i}}{g_{ref}}*a_{ref}$
$b_{i,low}=b_{ref}$
**end for**
Based on the $P1\left[DN_{low},DN_{high}\right]$, $P2\left[\bar{DN_{low}},\bar{DN_{high}}\right]$, and the correction model of the low-gain images:
$P1\left[DN_{low},DN_{high}\right]:\;DN_{high}=B_{0}+B_{1}*DN_{low}+B_{2}*DN_{low}{}^{2}+\ldots +B_{n}*DN_{low}{}^{n}$ $P2\left[\bar{DN_{low}},\bar{DN_{high}}\right]:\;\bar{DN_{high}}=B_{0}+B_{1}*\bar{DN_{low}}+B_{2}*{\bar{DN_{low}}}^{2}+\ldots +B_{n}*{\bar{DN_{low}}}^{n}$ $\bar{DN_{low}}=a_{i,low}*DN_{i,low}+b_{i,low}$
When the polynomial is of order n = 2, the relative correction model for high-gain images $P3\left[\bar{DN_{high}},DN_{high}\right]$ is:
$\begin{array}{c} \bar{DN_{high}}=\frac{{\left(B_{1}\pm \sqrt{-4B_{2}B_{0}+B_{1}^{2}+4B_{2}DN_{high}}\right)}^{2}a_{low}^{2}}{4B_{2}}\\ \;-\frac{\left(2B_{2}b_{low}a_{low}+B_{1}a_{low}\right)\left(B_{1}\pm \sqrt{-4B_{2}B_{0}+B_{1}^{2}+4B_{2}DN_{high}}\right)}{2B_{2}}+B_{2}b_{low}^{2}+B_{1}b_{low}+B_{0} \end{array}$
return $a_{i,low}$, $b_{i,low}$, and $P3\left[\bar{DN_{high}},DN_{high}\right]$.
